# Interleukin-6 in Anthracycline-Related Cardiac Dysfunction: A Comparison with Myeloperoxidase and TNF-Alpha

**DOI:** 10.3390/ijms26094071

**Published:** 2025-04-25

**Authors:** Anna Borowiec, Patrycja Ozdowska, Magdalena Rosinska, Agnieszka Maria Zebrowska, Sławomir Jasek, Beata Kotowicz, Agata Makowka, Joanna Waniewska, Hanna Kosela-Paterczyk, Elzbieta Lampka, Katarzyna Pogoda, Zbigniew Nowecki, Jan Walewski

**Affiliations:** 1 Non-Commercial Clinical Research Outpatient Clinic, Maria Sklodowska-Curie National Research Institute of Oncology, 02-781 Warsaw, Poland; 2 Department of Cancer & Cardio-Oncology Diagnostics, Maria Sklodowska-Curie National Research Institute of Oncology, 02-781 Warsaw, Poland; 3 Digital Medicine Center, Maria Sklodowska-Curie National Research Institute of Oncology, 02-781 Warsaw, Poland; 4 Unit for Screening Studies in Inherited Cardiovascular Diseases, The Cardinal Stefan Wyszynski National Institute of Cardiology, 04-628 Warsaw, Poland; 5 Cancer Biomarker and Cytokines Laboratory Unit, Maria Sklodowska-Curie National Research Institute of Oncology, 02-781 Warsaw, Poland; 6 Department of Radiology I, Maria Sklodowska-Curie National Research Institute of Oncology, 02-781 Warsaw, Poland; 7 Department of Soft Tissue, Bone Sarcoma and Melanoma, Maria Sklodowska-Curie National Research Institute of Oncology, 02-781 Warsaw, Poland; 8 Department of Lymphoid Malignancies, Maria Sklodowska-Curie National Research Institute of Oncology, 02-781 Warsaw, Poland; 9 Department of Brest Cancer and Reconstructive Surgery, Maria Sklodowska-Curie National Research Institute of Oncology, 02-781 Warsaw, Poland

**Keywords:** myeloperoxidase, Interleukin-6, TNF-alpha, cardiotoxicity, atherosclerosis, computed tomography, anthracycline, computed tomographic angiography, coronary artery calcium score, cancer therapy-related cardiovascular toxicity

## Abstract

Interleukin-6, myeloperoxidase, and tumor necrosis factor alpha are proinflammatory cytokines that play a role both in cardiovascular and oncological diseases. The study aimed to prospectively investigate the clinical value of interleukin-6, myeloperoxidase, and tumor necrosis factor alpha as potential biomarkers of cancer therapy-related cardiac dysfunction (CTRCD) in patients receiving anthracycline treatment. A total of 80 patients were included, with 77 (96.25%) followed for an average for 11.5 months. The mean age at baseline was 60.5 years, and 72 (93.51%) were women. Clinical risk factors were documented for all patients, and laboratory assessments, including measurements of IL-6, MPO, and TNF-α, were conducted. All participants also underwent echocardiography with assessment of global longitudinal strain (GLS). In the study group, coronary CT angiography with coronary artery calcium (CAC) score calculation was performed once at the beginning of the study. During observation, any degree of CTRCD was diagnosed in 48 (62.4%) patients. Mild CTRCD occurred in 38 (49.4%) patients, moderate CTRCD was diagnosed in 7 (9.1%), and severe in 3 (3.9%). In patients with high baseline risk, IL-6 levels were significantly elevated compared to those with moderate risk (*p* = 0.018). Higher levels of IL-6 were found to correlate with an increased grade of CTRCD. In a multivariate repeated measures model of the biomarkers studied, only a higher level of IL-6 was significantly associated with the diagnosis of CTRCD. Among the novel biomarkers studied, we found evidence for IL-6 for its potential use in the detection of cardiac dysfunction related to cancer therapy in patients treated with anthracyclines.

## 1. Introduction

Oncological treatment, including chemotherapy and radiotherapy, accelerates inflammatory degenerative processes, leading to the development of atherosclerosis and other cardiovascular complications [1,2,3,4,5]. In current cardio-oncological guidelines, cardiac biomarkers such as cardiac troponins (cTn) and natriuretic peptides (BNP, brain natriuretic peptide, and NT-proBNP, N-terminal pro-B-type natriuretic peptide) are recommended to monitor cardiac function during cancer treatment and diagnose cancer therapy-related cardiac dysfunction (CTRCD) [3]. However, their clinical utility remains debated. Since chronic inflammation can accelerate cardiovascular damage and promote fibrosis, atherosclerosis, adverse cardiac remodeling, and finally left ventricular dysfunction, it is reasonable to look for novel biomarkers that are involved in inflammatory and atherosclerotic pathways, which could be used to detect cardiotoxicity before it becomes irreversible. Interleukin-6 (IL-6) is a pro-inflammatory cytokine that plays an important role in the immune response and is considered an important inflammatory marker in the process of atherosclerosis [6,7]. Myeloperoxidase (MPO) is an enzyme secreted by leukocytes that is a marker of oxidative stress with proatherogenic and pro-oxidant properties, that was found elevated in chronic heart failure [8]. In some studies, IL-6 and MPO levels were elevated in response to chemotherapy-induced damage and were considered a marker of early left ventricular dysfunction [9]. Tumor necrosis factor alpha (TNF-α) is a pro-inflammatory cytokine that plays a key role in responses to inflammation and injury. It may contribute to vascular dysfunction, the development and progression of atherosclerosis, and adverse cardiac remodeling after myocardial infarction and heart failure [10].

The aim of this substudy of the ANTEC trial was to prospectively investigate the clinical value of IL-6, MPO, and TNF-alpha as potential biomarkers of CTRCD in patients receiving anthracycline treatment.

## 2. Results

### 2.1. Baseline Characteristics

A total of 80 patients were initially enrolled, with 77 (96.25%) completing the study. The mean follow-up duration was 11.5 months. The mean age at baseline was 60.5 years, with a majority of participants being women (72 patients, 93.51%). The most common cancer diagnoses were breast cancer (59 patients, 76.6%), followed by sarcoma (11 patients, 14.3%), and lymphoma (7 patients, 9.1%). The study population included patients without a prior diagnosis of heart failure or coronary artery disease. None of the patients had received chemotherapy before enrollment. According to the HFA-ICOS risk stratification for cancer therapy-related cardiovascular toxicity, 37 patients (48.1%) were classified as high-risk, while 40 patients (51.9%) were categorized as moderate-risk (Table 1).

### 2.2. Cardiovascular Outcomes

During observation, any degree of CTRCD was diagnosed in 48 (62.4%) patients. Mild CTRCD occurred in 38 (49.4%, 95% CI 38.2–60.5%) patients; moderate CTRCD was diagnosed in 7 (9.1%, 95% CI 4.3–18.0%); and severe in 3 (3.9%, 95% CI 1.2–11.6%). Additionally, 5 patients (6.49%) died during follow-up, with two deaths attributed to heart failure and three due to cancer progression. Of the 48 cases of CTRCD, 43 (93.8%) were diagnosed within the first 6 months of follow-up.

### 2.3. Association Between Novel Biomarkers and CAC Score or Coronary Artery Disease

**Figure 1:**
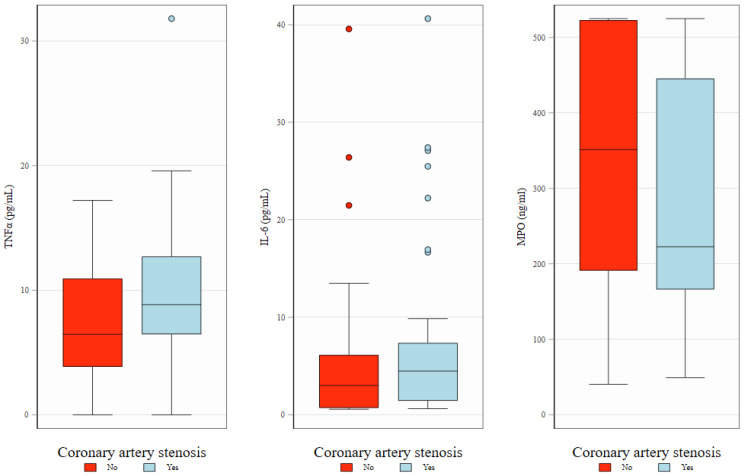
Baseline levels of TNFα, IL-6, and MPO in relation to the presence of coronary artery stenosis. Box plots illustrate the distribution of biomarkers—TNFα (**left**), IL-6 (**middle**), and MPO (**right**)—at baseline, stratified by the presence (red) or absence (blue) of coronary artery stenosis. The boxes represent interquartile interval with median marked as horizontal line. Whiskers and dots show, respectively, adjacent and outlying values.

There were no significant correlations between any of the biomarkers studied and the CAC score or the prevalence of coronary artery disease at baseline (visit 1), although a non-significant tendency for higher TNF-α level in individuals with coronary artery disease was observed, Figure 1.

### 2.4. Correlation Between Biomarkers at Subsequent Visits

**Figure 2:**
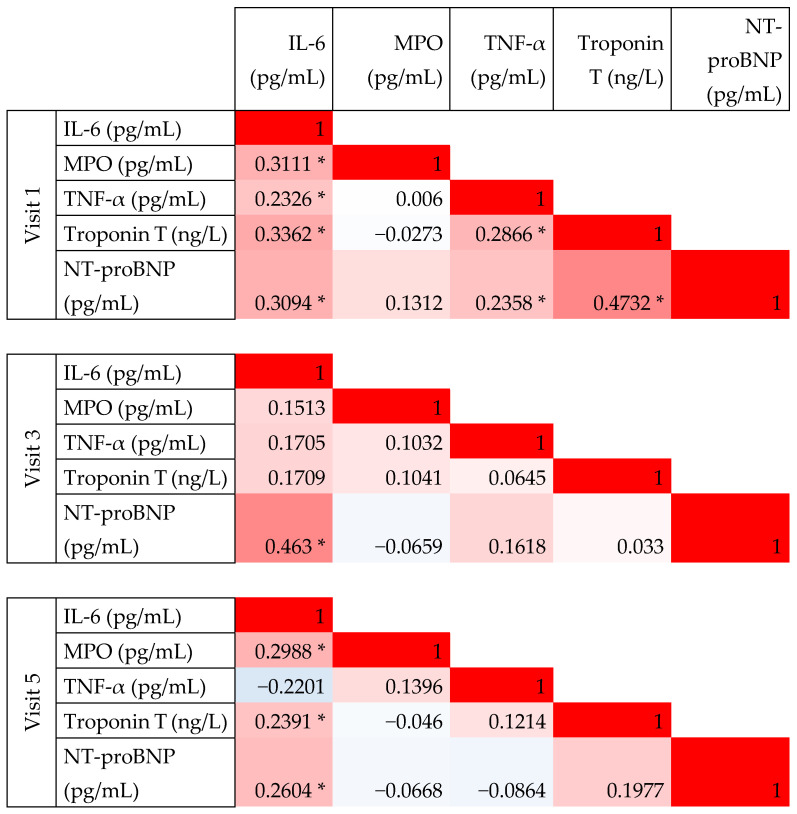
Correlation between novel biomarkers measured at baseline (Visit 1) and follow-up visits (Visit 3 and 5). This figure presents pairwise correlation coefficients; color scale is used from red to indicate perfect positive correlation (coefficient of 1), through white indicating no correlation (coefficient 0), and blue—perfect negative correlation (coefficient −1). Statistically significant coefficients (*p* < 0.05) are indicated with an asterisk.

**Figure 3:**
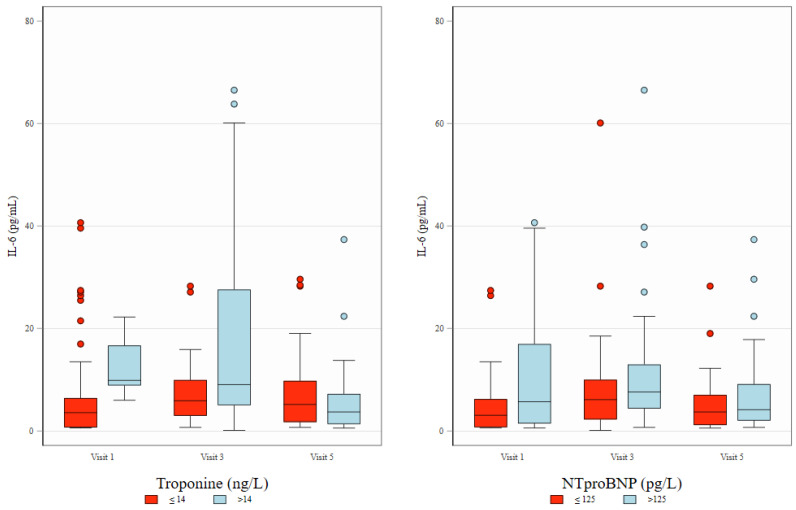
Distribution of IL-6 across study visits and its association with Troponin T and NT-proBNP. Box plots depict IL-6 concentrations (pg/mL) at three study visits (Visit 1, Visit 3, and Visit 5), stratified by levels of Troponin T (≤14 ng/L [normal] vs. >14 ng/L [elevated], left panel) and NT-proBNP (≤125 pg/L [normal] vs. >125 pg/L [elevated], right panel). The boxes represent interquartile interval with median marked as horizontal line. Whiskers and dots show, respectively, adjacent and outlying values.

At visit 1 (baseline, prior to anthracycline treatment), a significant weak to moderate correlation was observed between IL-6 and both TNF-α and MPO, with Spearman correlation coefficients (ρ) of 0.28 and 0.43, respectively. However, no significant correlation was found between TNF-α and MPO levels. At baseline, IL-6 and TNF-α levels also correlated with Troponin T and NT-proBNP levels. Notably, the significant correlations between IL-6 and both NT-proBNP and Troponin T were maintained throughout the follow-up period (Figure 2 and Figure 3).

Measurements of TNF-alfa and MPO during treatment (visits 3 and 5) did not show correlation with other biomarkers. Figure 3 presents the distribution of IL-6 levels across study visits and illustrates their association with Troponin T and NT-proBNP concentrations. Biomarker values throughout the observation visits are presented in Table 2.

### 2.5. Association Between Novel Biomarkers and CTRCD

**Figure 4:**
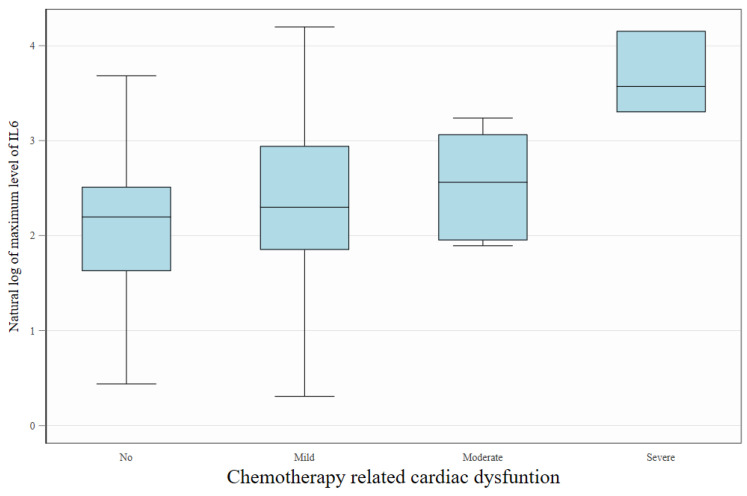
Association between IL-6 levels and the severity of cancer therapy-related cardiac dysfunction (CTRCD). Box plots display the natural logarithm of maximum during the follow-up IL-6 concentrations across increasing degrees of cardiac dysfunction severity (No, Mild, Moderate, Severe). Higher maximum IL-6 levels were observed in patients with more advanced CTRCD, suggesting a potential link between systemic inflammation and cardiotoxicity severity. The boxes represent interquartile interval with median marked as horizontal line. Whiskers show, adjacent and outlying values.

In patients with high baseline risk, IL-6 levels were significantly elevated compared to those with moderate risk (9.43 vs. 4.62 pg/mL, *p* = 0.018). Differences in baseline values of TNF and MPO between HR and MR groups were not significant (Table 2). During the anthracycline treatment, higher maximal levels of IL-6 were found to correlate with incidence of CTRCD (Figure 4). In a multivariate repeated measures model of the biomarkers studied, only a higher level of IL-6 was significantly associated with the diagnosis of CTRCD (Table 3).

The model fit indicates significant panel-level variance component (ρ = 0.94, *p*-value = 0.0112).

## 3. Discussion

This is the first study to prospectively evaluate the clinical value of novel biomarkers (IL-6, MPO, TNF-alpha) in cancer patients as potential biomarkers of cancer therapy-related cardiac dysfunction, defined according to current ESC cardio-oncology guidelines. Among the biomarkers studied, we found evidence only for IL-6 for its potential use in the detection of cardiac dysfunction related to cancer therapy in patients treated with anthracyclines.

In the study by Lakhani et al. in breast cancer patients, MPO levels were significantly elevated 3 and 6 months after the initiation of anthracyclines compared to the control. Furthermore, their results also showed significantly higher levels of IL-6 at 6 months compared to the control and baseline. Moreover, high cardiac troponin T was significantly correlated with MPO and IL-6 [11]. In our study, a significant increase in IL-6 levels was observed at 6 months, whereas MPO and TNF-alpha did not show any significant changes during observation. In another study, serum levels of IL-6 increased significantly in the placebo arm compared to baseline, but remained unchanged in the telmisartan arm in epirubicin-treated oncological patients [12]. There were several studies that sought novel biomarkers in cardiotoxicity. Unfortunately, pro-inflammatory cytokines, such as IL-6 and TNF-alpha, were rarely involved in these studies, contrary to MPO, which was studied more frequently and in most studies was considered an important novel biomarker. In the multiple biomarker study (TnI, CRP, GDF-15, MPO, PlGF, and sFlt-1) by Ky et al., only early increases in TnI and MPO levels were associated with subsequent cardiotoxicity in patients with breast cancer treated with doxorubicin, taxanes, and trastuzumab. Additionally, the combination of these two biomarkers provided additive information on the risk of developing cardiotoxicity [13]. In the study by Putt et al., among the biomarkers studied (hs-cTnI, hsCRP, NT-proBNP, GDF-15, MPO, PlGF, sFlt-1, gal-3) only an increase in MPO were associated with cardiotoxicity throughout the course of doxorubicin and trastuzumab therapy [14]. In contrast, in our study, where patients were treated with doxorubicin but not trastuzumab, no increase in MPO was observed during the observation. In another study of multiple biomarkers by Todorowa et al., plasma samples for biomarkers of inflammation, hypercoagulability, and endothelial dysfunction—including C-reactive protein (CRP), thrombomodulin (TM), thrombin-antithrombin complex (TAT), MPO, von Willebrand factor (vWF), and P-selectin—were examined. During observation, patients with abnormal decrease in left ventricle ejection fraction (LVEF) >10% had significantly elevated levels of MPO and TM both at baseline and after the first dose of chemotherapy based on doxorubicine compared to patients with normal LVEF after adjustment for race, age, BMI, and biological subtype of breast cancer [9]. In the study by Demissei et al., repeated measures of high-sensitivity cardiac troponin T (hscTnT), NT proBNP (N-terminal proB-type natriuretic peptide), myeloperoxidase, placental growth factor, and growth differentiation factor 15 were evaluated in a cohort of 323 patients treated with anthracyclines and/or trastuzumab. Among the newer biomarkers, increases in MPO were associated with an increased risk of cardiac dysfunction in anthracycline-based treatment groups [15]. In the meta-analysis by Wu et al., MPO levels were not only associated with an increased risk of cardiotoxicity, but were also a more effective indicator of response to cancer treatment compared to levels of TnI and NT-proBNP [16]. The opposite was found in our study: there was no significant correlation between MPO and CTRCD development. Unlike our observations, in the Camilli et al. study, no correlation was observed between changes in LVEF or GLS and IL6 in oncological patients. However, their population was treated with CAR-T cell therapy, and our population was treated with anthracyclines [17].

### Strengths and Limitations

A major strength of this study is the prospective design and comprehensive evaluation of biomarkers alongside echocardiographic and clinical data. However, certain limitations must be acknowledged. The population included a small cohort from a single cancer center, and the small sample size may have limited the power of this study. Furthermore, the study population consisted almost exclusively of women, since most of the patients had breast cancer. Therefore, the results cannot be extrapolated to the general population.

## 4. Materials and Methods

### 4.1. Study Structure

The ANTEC study is a single center prospective observational trial that, as an exploratory objective, evaluates the association of IL-6, MPO, and TNF-alpha with the risk and diagnosis of CTRCD related to chemotherapy based on anthracycline. The trial was carried out according to the principles of the Declaration of Helsinki and the guidelines of the International Conference on Harmonization Good Clinical Practice. The conduct of the study was approved by an independent Ethics Committee at the Maria Sklodowska-Curie National Research Institute of Oncology in Warsaw (date of approval: 28 October 2021, approval no. 80/2021), and all participants gave their informed written consent before entering the study. The registration identifier on clinicaltrials.gov is NCT05118178. The study included 80 cancer patients, diagnosed and qualified for systemic treatment with anthracycline chemotherapy at the Maria Sklodowska-Curie National Research Institute of Oncology in Warsaw. The study protocol with inclusion and exclusion criteria, schedule visits, and procedures was previously published [18]. None of the patients had previously received any type of chemotherapy or radiotherapy. None of the patients had been diagnosed with coronary artery disease or heart failure at baseline.

### 4.2. Patients Classification, CCTA, and Echocardiography

The study investigators collected clinical variables based on the medical documentation provided by the patient and medical anamnesis. In all patients, coronary CT angiography (CCTA) was performed once at the beginning of the study. Coronary artery calcium (CAC) score was calculated according to the Agatston method. The area of calcified atherosclerosis was multiplied by a density weighting factor for each slice and summed across the entire coronary arterial tree. Contrast coronary CTA was performed using a 64-slice multidetector CT scanner (Revolution Evo, GE Healthcare, Waukesha, WI, USA) with prospective ECG triggering. Scans were performed in accordance with the Society of Cardiovascular Computed Tomography guidelines in order to achieve high image quality with the lowest possible patient radiation and contrast dose. The presence, extent and severity of coronary artery stenosis was defined as minimal (<25%), mild (25 to 49%), moderate (50 to 69%), severe (70 to 99%), and occluded (100%), according to the guidelines [19]. Transthoracic echocardiography was performed using a Philips EPIQ Elite instrument (Philips Healthcare, Andover, MA, USA). The following echocardiographic parameters were evaluated: ventricular diameters, diastolic and systolic functions, and two-dimensional left ventricular peak systolic global longitudinal strain (GLS), which was analyzed using a semiautomated speckle tracking imaging technique from the three standard apical views. Analyses were performed by a board certified physician who was blinded to all clinical characteristics. Blood samples were collected and analyzed by the central laboratory (The Maria Sklodowska-Curie National Research Institute of Oncology). Laboratory tests included, among others, creatinine, glucose, complete blood count, glycated hemoglobin, lipid profile, plasma levels of troponin T (TnT), and N-terminal pro-B-type natriuretic peptide (NT-proBNP).

### 4.3. Blood Samples for IL-6, MPO, and TNF-Alpha

Blood was collected from patients at baseline (visit 1), six months (visit 3), and 12 months (visit 5) and then centrifuged at 2600 rpm at 15 min. After centrifugation, serum samples were obtained and stored in low temperature freezers (−80 °C) in aliquots until analysis time. IL-6, MPO, and TNF-alpha concentrations were measured by means of ELISA kits (R&D Systems, Bio-Techne, Minneapolis, MN, USA). This assay works according to the principle of the quantitative sandwich enzyme immunoassay technique. The concentrations of the parameters were determined by measuring optical density on a spectrophotometer, at a wavelength of 450 nm. The sensitivity of the assays was: IL-6: 0.368 pg/mL, MPO: 0.014 ng/mL, and TNF-alpha: 4.0 pg/mL.

### 4.4. Data Acquisition and Statistical Analysis

#### 4.4.1. Data Acquisition and Quality Control

All measurements were performed according to routine diagnostic standards. Critical measurements (CCTA, echocardiography, biomarkers) were reviewed and coded by a trained member of the study team to ensure the comparability of the results. Clinical data were captured by manual entry into electronic case report forms (eCRF) and laboratory data extracted from the Hospital Information System (HIS). The data were validated by the clinical team to eliminate inconsistent, missing, and outlying values.

#### 4.4.2. Sample Size Calculations and Statistical Analysis

The sample size was calculated based on the primary objective of the ANTEC study, which is the hypothesis that the incidence of CTRCD will increase twofold, from 30–40%, in the presence of coronary artery stenosis, the prevalence of which was estimated at 30–40% of the patient population [11]. Therefore, the current biomarker analysis remains exploratory. To explore the correlation between examined parameters and already established biomarkers, we calculated the Spearman correlation at baseline as well as the correlations during treatment using data from Visits 3 and 5 and repeated measures of correlation coefficients. To understand the potential role of biomarkers as predictors of CTRCD, we analyzed the level of biomarkers at Visit 1 (before treatment). In addition, we investigated the association of the level of biomarkers measured at the same time as the status of CTRCD was evaluated using multivariable random effect logistic regression with CTRCD status (yes/no) as the outcome variable. Data after the diagnosis of CTRCD were excluded due to possible effect of the toxicity management on the measurements. Fractional polynomial models were used for finding the best fitting transformations of these predictors, however yielding no improvement over linear fit. Significant results are reported at the alfa = 0.05 level. Statistical analysis was performed in Stata/SE 17.0 (StataCorp LCC, College Station, TX, USA).

## 5. Conclusions

Among the novel biomarkers studied, we found evidence for IL-6 for its potential use in the detection of cardiac dysfunction related to cancer therapy in patients treated with anthracyclines. In addition to its clinical application in cardiotoxicity, the findings suggest the potential of anti-IL-6 therapeutics to effectively target both cardiovascular and oncological diseases. Furthermore, adopting a multibiomarker approach may enhance the sensitivity of predicting the risk of cardiac dysfunction in patients undergoing anthracycline therapy.

## Figures and Tables

**Table 1:** Baseline characteristics of the study group (N = 77).

Patients Characteristics	Value
Sex, *n* (%)	Male: 5 (6.5%)
	Female: 72 (93.5%)
Age group, *n* (%)	<65 years: 41 (53.2%)
	≥65 years: 36 (46.8%)
Age (years)	Mean (SD): 60.5 (12.2)
	Median [min–max]: 64 [24–80]
Cancer type, *n* (%)	Breast: 59 (76.6%)
	Lymphoma: 7 (9.1%)
	Sarcoma: 11 (14.3%)
Risk group according to HFA-ICOS, *n* (%)	High Risk: 37 (48.1%)
	Moderate Risk: 40 (51.9%)
BMI, *n* (%)	<25: 24 (31.2%)
	25–<30: 28 (36.4%)
	≥30: 25 (32.5%)
Coexisting conditions *, *n* (%)	Hypertension: 60 (77.9%)
	Hyperlipidemia: 58 (75.3%)
	Diabetes: 11 (14.3%)
	Chronic kidney disease: 12 (15.6%)
	Ever smoking: 37 (48.1%)
NYHA scale, *n* (%)	1: 54 (70.1%)
	2: 23 (29.9%)
ECOG score, *n* (%)	0: 55 (71.4%)
	1: 21 (27.3%)
	2: 1 (1.3%)
Medications, *n* (%)	Beta-blockers: 33 (42.9%)
	ACE-i: 34 (44.2%)
	Statins: 23 (29.9%)
Coronary artery stenosis, *n* (%)	No stenosis: 36 (46.8%)
	Minimal (<25%): 7 (9.1%)
	Mild (25–49%): 19 (24.7%)
	Moderate (50–69%): 6 (7.8%)
	Severe (70–99%): 9 (11.7%)
Calcium score in cardiac CT scan, *n* (%)	0: 36 (46.8%)
	1–99: 24 (31.2%)
	100–399: 14 (18.2%)
	400+: 3 (3.9%)
CAC score	Mean (SD): 84.4 (173.6)
	Median [min–max]: 4 [0–1041]
Anthracycline dose (mg/m^2^), *n* (%)	<250: 62 (80.5%)
	≥250: 15 (19.5%)
Troponin T, *n* (%)	≤14 ng/L: 71 (92.2%)
	>14 ng/L: 6 (7.8%)
Troponin T (ng/L)	Mean (SD): 8 (3.8)
	Median [min–max]: 7 [3–21]
NT-proBNP, *n* (%)	≤125 pg/mL: 48 (62.3%)
	>125 pg/mL: 29 (37.7%)
NT-proBNP (pg/mL)	Mean (SD): 137.9 (142)
	Median [min–max]: 94 [10–629]
LV GLS (%)	Mean (SD): 19.1 (2)
	Median [min–max]:19.2 [14.2–25.1]
LV EF (%)	Mean (SD): 61.6 (3.5)
	Median [min–max]: 62 [50–69]
Creatinine (mg/dL)	Mean (SD): 0.8 (0.2)
	Median [min–max]: 0.8 [0.4–1.6]
EGFR (mL/min/1.73 m^2^)	Mean (SD): 79.5 (21.7)
	Median [min–max]: 76 [30–146]
Triglycerides (mg/dL)	Mean (SD): 118.7 (50.5)
	Median [min–max]: 105 [43.6–251]
HDL (mg/dL)	Mean (SD): 58.8 (15.8)
	Median [min–max]: 57 [30–119]
LDL (mg/dL)	Mean (SD): 130.9 (37)
	Median [min–max]: 126 [62.9–226]
Glycated hemoglobin (%)	Mean (SD): 5.8 (1)
	Median [min–max]: 5.8 [0–9.8]
Biomarkers	
TNF-α (pg/mL)	Mean (SD): 8.7 (5.5)
	Median [min–max]: 8 [0–31.8]
MPO (pg/mL)	Mean (SD): 312.9 (163.3)
	Median [min–max]: 309.5 [40.8–525]
IL-6 (pg/mL)	Mean (SD): 6.8 (8.8)
	Median [min–max]: 3.8 [0.6–40.6]

* categories may overlap.

**Table 2:** Biomarker values throughout the observation visits, by HFA-ICOS risk group evaluated at baseline.

		High Risk	Medium Risk	Total	*p*-Value *
		Mean (sd), Median [Min–Max]	Mean (sd), Median [Min–Max]	Mean (sd), Median [Min–Max]	
Visit 1 (baseline)	IL-6 (pg/mL)	9.43 (10.97). 5.98 [0.59–40.62]	4.62 (5.78). 2.31 [0.7–26.39]	6.83 (8.83). 3.79 [0.59–40.62]	0.0186
	MPO (pg/mL)	327.45 (163.98). 336.7 [49.4–525]	300.55 (163.69). 279.02 [40.75–525]	312.91 (163.26). 309.5 [40.75–525]	0.4019
	TNF-α (pg/mL)	9.88 (6.32). 8.67 [0–31.8]	7.7 (4.52). 6.85 [0–17.2]	8.7 (5.49). 7.95 [0–31.8]	0.1446
Visit 3	IL-6 (pg/mL)	16.23 (19.25). 8.28 [0.7–66.5]	7.98 (7.56). 5.94 [0.12–36.37]	11.91 (14.83). 6.87 [0.12–66.5]	0.18877
	MPO (pg/mL)	214.3 (143.08). 189.63 [31.3–525]	185.19 (137.46). 122.5 [45.8–525]	199.05 (139.8). 154.6 [31.3–525]	0.5272
	TNF-α (pg/mL)	6.77 (6.23). 6.59 [0–20.3]	5.38 (7.66). 3.8 [0–34.2]	6.04 (7). 4.5 [0–34.2]	0.1089
Visit 5	IL-6 (pg/mL)	7.62 (8.38). 5.43 [0.7–37.33]	6.4 (7.08). 3.28 [0.56–29.58]	6.94 (7.65). 4.13 [0.56–37.33]	0.3985
	MPO (pg/mL)	209.77 (123.58). 203.75 [44.65–525]	214.9 (147.25). 184.13 [21.3–525]	212.62 (136.32). 193.93 [21.3–525]	0.8415
	TNF-α (pg/mL)	2.95 (4.87). 0 [0–20.54]	4.48 (6.25). 1.35 [0–26.23]	3.8 (5.69). 0 [0–26.23]	0.2929

* Wilcoxon rank-sum test comparing respective biomarker levels between high and medium risk groups.

**Table 3:** Association between biomarkers and CTRCD in multivariate random-effect logistic regression.

		Odds Ratio	*p*-Value
Baseline values			
IL-6 (pg/mL)	Increase by 1 pg/mL	0.64 (0.27–1.53)	0.316
TNF-α (pg/mL)	Increase by 1 pg/mL	0.88 (0.38–2.02)	0.756
MPO (ng/mL)	Increase by 1 ng/mL	1 (0.97–1.03)	0.894
Values observed during treatment			
IL-6 (pg/mL)	Increase by 1 pg/mL	1.52 (1.05–2.22)	0.028
TNF-α (pg/mL)	Increase by 1 pg/mL	1.16 (0.76–1.78)	0.486
MPO (ng/mL)	Increase by 1 ng/mL	0.99 (0.97–1.01)	0.309

## Data Availability

The datasets used and/or analyzed during the current study are available from the corresponding author on reasonable request.

## Abbreviations

CTRCD	cancer therapy-related cardiac dysfunction
CTR-CVT	cancer therapy-related cardiovascular toxicity
HFA-ICOS	Heart Failure Association-International Cardio-Oncology Society
CCTA	Coronary computed tomography angiography
CAC score	coronary artery calcium score
GLS	left ventricular peak systolic global longitudinal strain
hs-cTnT	high-sensitivity cardiac troponin T
NT-proBNP	N-terminal pro-B-type natriuretic peptide
eCRF	electronic case report forms
HIS	Hospital Information System
LVEF	left ventricle ejection fraction
CVD	cardiovascular disease
IL-6	interleukin-6
MPO	myeloperoxidase
TNF-α	tumor necrosis factor alpha
